# Integration of UV Stability and Shelf-Life Prediction in a Colorimetric Intelligent Label for Real-Time Monitoring of Shrimp Freshness

**DOI:** 10.3390/foods15081388

**Published:** 2026-04-16

**Authors:** Xiujin Chen, Shiqiang Yu, Yang Qu, Jing Wang, Minghui Dai, Weiguo Song, Peihong Liu, Yujuan Suo

**Affiliations:** 1 Henan International Joint Laboratory of Green Food Processing and Quality and Safety Control, College of Food and Bioengineering, Henan University of Science and Technology, Luoyang 471000, China; chenxiujin9610@126.com (X.C.);; 2 Institute for Agro-Food Standards and Testing Technology, Laboratory of Quality & Safety Risk Assessment for Agro-Products (Shanghai), Ministry of Agriculture and Rural Affairs, Shanghai Academy of Agricultural Sciences, Shanghai 201403, China

**Keywords:** intelligent labels, UV shielding, anthocyanins, carbon-coated nano-TiO_2_, remaining shelf-life

## Abstract

The instability of pigments and non-quantitative indication limit the application of intelligent labels in food freshness monitoring. Natural anthocyanins face challenges including photodegradation and difficulty in quantifying shrimp freshness. To solve these problems, this study prepared a colorimetric intelligent label with UV-shielding and real-time monitoring functions. Carbon-coated nano-TiO_2_ (C-TiO_2_) was synthesized by the hydrothermal method and combined with blueberry anthocyanins (BAs) in an agarose (AG)/gellan gum (GG)/glycerol matrix. The label properties were characterized and a remaining shelf-life prediction model was established based on the correlation between label color difference (ΔE) and shrimp total volatile basic nitrogen (TVB-N). The results demonstrated that C-TiO_2_ could enhance compatibility and color stability, and maintain mechanical properties. After 24 h of ultraviolet irradiation, the BA degradation rate was 98.4% in the GAB group and 62.8% in the GABT-0.05 group, representing a reduction of 35.6% compared to the former. This indicates that the addition of C-TiO_2_ significantly enhanced photostability. The predictive model demonstrated an error below 10% at both 10 °C and 20 °C conditions, indicating its potential for shelf-life prediction applications. This dual-functional label provides a reliable method for visual and quantitative evaluation of shrimp freshness.

## 1. Introduction

Shrimp, as a high-nutrition aquatic product with high water content and easy spoilage, is one of the most consumed seafoods worldwide. The preservation and real-time freshness monitoring of shrimp are crucial for controlling product quality, reducing postharvest loss, and safeguarding consumer food safety [1]. Colorimetric intelligent labels have emerged as a focal point in food freshness monitoring due to their operational simplicity and intuitive visual outputs [2,3]. These labels function by leveraging the chromatic response of pigments to pH shifts induced by specific volatile compounds, such as TVB-N, carbon dioxide, and hydrogen sulfide, produced during the storage of perishable foods, and thereby enable direct, non-invasive real-time freshness assessments [4,5]. The integration of UV shielding performance and shelf-life prediction function into colorimetric intelligent labels can realize the dual goals of improving the stability of sensing pigments and quantifying food freshness, and the UV-associated colorimetric measurements provide a reliable technical approach for real-time monitoring of shrimp freshness and shelf life. Among natural pigments, anthocyanins, which are pH-sensitive flavonoids, are particularly promising as sensing materials for smart food packaging due to their exceptional safety, biocompatibility, and vivid color transition characteristics [6,7,8]. Traditional shrimp freshness monitoring methods, such as laboratory-based TVB-N determination, microbial count detection, and sensory evaluation, are constrained by time-consuming operations, high testing costs, and destructive sampling, which cannot meet the demand for real-time and in situ monitoring in the aquatic product supply chain [9]. Therefore, developing real-time freshness monitoring technology using intelligent labels has the potential to improve detection efficiency and reduce production and circulation costs, and the research on accurate and efficient intelligent labels is of great practical significance for the development of the aquatic product industry. Despite these advantages, the practical deployment of chromogenic reaction systems faces two critical challenges. First, photostability issues arise under UV exposure, where UV absorption excites chromophoric groups, such as pyrylium rings and phenolic hydroxyls. This excitation can lead to irreversible bond cleavage or rearrangement, resulting in a degradation of functionality, with losses of up to 63% [10,11]. Second, the absence of quantitative indications limits most existing systems to visual cues, without establishing a quantitative correlation to actual freshness parameters or shelf-life predictions. This limitation restricts their utility in data-driven supply chain decisions.

In research on enhancing natural pigment stability, nano-TiO_2_ has attracted much attention due to its excellent UV absorption and high chemical stability [12]. Traditional anthocyanin stabilization strategies (copigmentation, polysaccharide encapsulation, nano-ZnO/SiO_2_ doping) have certain photostability-improving effects but inherent limitations: copigmentation and polysaccharide encapsulation cannot fundamentally block UV radiation, only delaying degradation [13]; and conventional nano-TiO_2_ and nano-ZnO/SiO_2_ easily generate reactive oxygen species (ROS) under UV irradiation due to photocatalytic activity [14,15], accelerating anthocyanin oxidative degradation and offsetting UV shielding effects. To solve this contradiction, surface carbon coating of nano-TiO_2_ has become a new modification strategy with dual advantages of efficient UV shielding and photocatalytic inhibition [16,17,18,19,20]; the carbon layer acts as a physical barrier to weaken UV excitation and blocks ROS formation by inhibiting photogenerated electron–hole pair separation [21,22,23], breaking the bottleneck of traditional nano-stabilizers and accelerating anthocyanin degradation while shielding UV. Regarding food safety, the U.S. FDA has approved nano-TiO_2_ for food packaging materials (≤25% of food-contact material weight) [14], and Li et al. reported that CQD modification did not increase TiO_2_ safety risks [24]. However, no literature has reported the application of carbon-coated nano-TiO_2_ in intelligent label preparation to date.

Quantitative shelf-life prediction for fresh agricultural products is a key research focus, but there is an inherent contradiction between model accuracy and key parameter availability. Kinetic models (e.g., Arrhenius) relying on precise biochemical indicators (e.g., microbial counts, volatile metabolites) achieve high accuracy but require destructive sampling and complex analysis [25], so are unsuitable for real-time supply chain monitoring [26]. Models based on easily obtainable parameters (e.g., color, texture images, intelligent label color changes) improve convenience but have limited accuracy due to nonlinear relationships between color and spoilage indicators and lack of links to biochemical mechanisms [27]. TVB-N-responsive intelligent labels, which connect observable color parameters with spoilage processes, provide a new approach to balance availability and accuracy [28], yet there are no systematic reports on using their color data to construct quantitative shelf-life prediction models for shrimp preservation.

To address these challenges, this study designed and fabricated a dual-functional intelligent label. The label enhances UV stability by incorporating C-TiO_2_ and establishes a shelf-life prediction model based on TVB-N response color, enabling real-time, quantitative monitoring of shrimp freshness and remaining shelf life, thereby providing a novel solution for smart packaging applications.

## 2. Materials and Methods

### 2.1. Materials

Fresh live shrimp (scientific name: *Penaeus vanname*; specifications/weight: body length 10–12 cm, individual weight 15–20 g; initial condition: fresh shrimp, transported back to the laboratory within 0.5 h after purchase, cleaned and used immediately) were purchased from a local supermarket in Fengxian, Shanghai, China. Agarose (AG) was acquired from Shanghai Sangon Biotech Co., Ltd. (Shanghai, China). Gellan gum (GG), blueberry anthocyanins (BAs), nano-TiO_2_ (particle size: 21 nm, purity: 99.5%), glycerol, methylene blue (MB), and glucose were all purchased from National Medicines Chemical Reagent Co., Ltd. (Shanghai, China). All chemicals were of analytical grade.

### 2.2. Labels Preparation

C-TiO_2_ was prepared using the method described by Ali et al. with minor modifications [29]. Specifically, 1.0 g of nano-TiO_2_ was dispersed in 50 mL of deionized water under ultrasonic treatment (300 W, 30 min). Following this, 1.0, 2.0, and 3.0 g of glucose (AR grade) were added with magnetic stirring, and the mixture was transferred to a 100 mL Teflon-lined autoclave for hydrothermal reaction at 180 °C for 12 h. After natural cooling, the precipitate was collected by centrifugation, washed three times with deionized water, and vacuum-dried at 60 °C for 12 h. Carbon-coated nanomaterials labeled 1C-TiO_2_, 2C-TiO_2_, and 3C-TiO_2_ were obtained after calcination at 500 °C under a nitrogen atmosphere (heating rate: 5 °C/min).

Functional label preparation (Figure S1): A quantity of 1 g of AG was added to 100 mL of deionized water and continuously stirred at 100 °C until completely dissolved to form a homogeneous solution. Subsequently, 2.0 g of glycerol and 1.0 g of GG were added under heating with stirring until full dissolved, yielding GA solution. Subsequently, C-TiO_2_ was uniformly dispersed in 2.0 g of glycerol. After 20 min of ultrasonic treatment, it was mixed with GA solution without glycerol to achieve final concentrations of 0.05 wt% and 0.10 wt% in the system. The mixture was stirred at 60 °C for 30 min, followed by addition of 25.0 mg of BA. Degassing was performed through 10 min ultrasonic treatment, resulting in GABT-0.05 or GABT-0.1 composite solutions (named according to the mass of 2C-TiO_2_ used). Finally, 15.0 mL of each solution was poured into 90.0 mm diameter glass Petri dishes and dried in a 40 °C hot-air oven for 10–12 h to fabricate free-standing labels. The obtained labels were vacuum-sealed and stored in a light-proof environment.

### 2.3. Characterization of the Labels

The characterization of the labels was conducted through multiple integrated methods (all characterization experiments were performed with three parallel replicates). Microscopic morphology was observed using a scanning electron microscope (SEM, Apreo 2S HiVac, Thermo Fisher Scientific, Waltham, MA, USA) at an accelerating voltage of 5 kV after freeze-drying with liquid nitrogen and gold coating. Chemical structure was analyzed by Fourier transform infrared spectroscopy (FTIR, Nicolet Is5, Thermo Fisher Scientific, USA) in the spectral range of 4000–400 cm^−1^ with a resolution of 4 cm^−1^. Crystalline structure was characterized via X-ray diffraction (XRD, Bruker D8 Advance, Bruker Corporation, Billerica, MA, USA) in the 2θ range of 5–45° at a scanning speed of 0.1°/s to measure crystallinity and record diffraction patterns. Thermal properties were determined by recording thermogravimetric analysis (TGA) curves using a thermal analyzer (TG209 F3, Netzsch, Selb, Germany) under N_2_ atmosphere, heating from 30 °C (room temperature) to 800 °C at a rate of 10 °C/min, and a sample weight of 5 ± 0.1 mg. Light transmission was evaluated through UV-Vis transmittance spectra in the wavelength range of 200–800 nm using a UV-Vis spectrophotometer (UV-2355, UNICO, Shanghai, China). Thickness was measured using a micrometer screw, with three repeated measurements. Mechanical properties including tensile strength (TS) and elongation at break (EAB) were determined according to American Standard ASTM D882 [30] using a universal testing machine (JD-D500, Jindun Electronic Technology Co., Ltd., Dongguan, China); the sample dimensions were 50 mm × 10 mm, with testing conditions of 25 °C and 50% relative humidity (RH), and a crosshead speed of 50 mm/min. Moisture content (MC) was calculated from the mass difference before and after drying the label at 105 °C to constant weight, while water solubility (WS) was calculated from the mass difference between two drying stages after soaking the dried label in deionized water for 24 h and re-drying at 105 °C to constant weight. Water vapor permeability (WVP) was measured following Rong’s method (Equation (1)) by placing anhydrous CaCl_2_ (dried at 200 °C for 2 h and cooled) in weighing bottles (1 cm below the rim) [31], covering the bottle mouth with the label and sealing with Vaseline, recording initial mass, and weighing daily for 8 days in a desiccator containing deionized water (100% RH) at 25 °C. Equation (1) is as follows, where Δ*m* represents mass change (g), *d* denotes label thickness (m), *A* indicates moisture permeable area (m^2^), *t* stands for time (s), and Δ*p* signifies vapor pressure difference (Pa).

(1) $$\begin{array}{c} WVP\left(\times 10^{-9}{\mathrm{gm}}^{-1}{\mathrm{Pa}}^{-1}s^{-1}\right)=\frac{∆m\times d}{A\times t\times ∆p} \end{array}$$

### 2.4. Analysis of Color Stability and pH Sensitivity of Labels

Samples of GAB, GABT-0.1, GABT-0.05, and GA were cut into equal-area (1 cm^2^) pieces, exposed to UVA/UVB (the lamp type was a UVA/UVB composite ultraviolet lamp (315–400 nm UVA, 280–315 nm UVB), with a sample–lamp distance of 15.0 cm. Temperature control during irradiation was maintained at 25 ± 1 °C and simulated light (0.5 W/m^2^) for 0 h, 6 h, 12 h, and 24 h with three parallel samples per condition, ground, extracted with 5 mL acidic ethanol (ethanol:0.1 M HCl = 85:15, *v*/*v*) under dark agitation for 1 h, centrifuged; the extraction was repeated twice. Combined supernatants were diluted to 25.0 mL in a brown volumetric flask, BA concentration determined via the pH-differential method with degradation rate calculated for UV stability evaluation. Simultaneously, label samples stored at 4 °C and 25 °C for one week had ΔE (Equation (2)) measured every 24 h using a colorimeter (CM-A298, Konica Minolta, Tokyo, Japan) following Rong’s method for color stability assessment, while pH sensitivity was characterized by observing color changes when labels were immersed in pH 3–12 buffer solutions [31]. The buffer solution used was Britton–Robinson universal buffer (pH 3–12). The labels were immersed in each pH buffer for 3 min and allowed to equilibrate for 10 min before observing color changes and measuring ΔE. Δ*E* is calculated from the differences in L* (lightness), a* (green–red deviation), and b* (blue–yellow deviation) using Equation (2).

(2) $$\begin{array}{c} ∆E=\sqrt[{2}]{({∆L*)}^{2}+{(∆a*)}^{2}+({∆b*)}^{2}} \end{array}$$

### 2.5. Establishment of Shrimp Shelf-Life Prediction Model

Using the key indicator for characterizing the freshness of aquatic products, TVB-N, as the core parameter, a shelf-life prediction model for shrimp under various temperature conditions was established [32]. Initially, fresh shrimp samples are placed in sterile Petri dishes and stored in constant-temperature incubators at 0 °C, 4 °C, 12 °C, 20 °C, and 28 °C for gradient storage. Temperature sampling times were 0 °C (0, 24, 48, 72, 96, 120, 144, 168, 192, 216, 240 h), 4 °C (0, 24, 48, 72, 96, 120, 144, 168, 192 h), 12 °C (0, 9, 18, 27, 36, 45, 54, 63 h), 20 °C (0, 6, 12, 18, 24, 30, 36, 42 h), and 28 °C (0, 4, 8, 12, 16, 20, 24 h). The TVB-N content is measured at predetermined time intervals according to the method specified in the Chinese National Standard GB 5009.228-2016 [33], with three parallel samples set for each temperature condition to ensure data reliability. The TVB-N accumulation rate of shrimp exhibits first-order reaction kinetics characteristics with exponential growth, whereas zero-order or second-order models cannot accurately describe this process. Subsequently, a first-order kinetic model is applied to fit the TVB-N accumulation process at each temperature, allowing for the determination of reaction rate constants for different temperatures [34]. The initial TVB-N value A_0_ was 1.39 ± 0.06 mg/100 g (consistent across all temperature groups). The Arrhenius equation is then employed to establish a quantitative relationship between the rate constants and temperature, facilitating the calculation of the apparent activation energy and pre-exponential factor. Finally, by setting 30 mg/100 g as the spoilage threshold based on relevant literature [35], the kinetic equation and Arrhenius equation are combined to derive the shelf-life prediction model.

### 2.6. Application of Labels as Colorimetric Indicators

Fresh shrimp samples of uniform specifications were placed in sterile Petri dishes (90 mm in diameter) with a label (15 mm in diameter) fixed on the inner side of the Petri dish lid. The shrimp mass in each petri dish was 32 g ± 1 g, with an effective headspace volume of 60 mL. The edges of the Petri dishes were sealed with Parafilm film to create a sealed environment and stored in a constant-temperature incubator at 25 ± 0.5 °C. Samples were collected every 4 h, and the TVB-N content was determined using the micro-diffusion method according to the Chinese National Standard GB 5009.228-2016. Simultaneously, the ΔE value (calculated from the L, a, and b parameters) was measured using a colorimeter. The correlation between TVB-N and ΔE was analyzed using the Pearson correlation coefficient, and all experiments were performed with three independent replicates.

### 2.7. Validation of the Accuracy of Quantitative Shelf-Life Indicated on Labels

Model accuracy was quantitatively evaluated by calculating the mean absolute error (MAE), root mean square error (RMSE), and relative error (*RE*, Equation (3)) between predicted values (*A_pre_*) and observed values (*A_obs_*) under 4 °C, 10 °C and 20 °C conditions. *A_pre_* represents the predicted value of the model; *A_obs_* represents the remaining shelf-life measured value of the experiment. RE was determined using Equation (3).

(3) $$\begin{array}{c} RE=\frac{\left|A_{\mathrm{pre}}-A_{\mathrm{obs}}\right|}{A_{\mathrm{obs}}}\;\times \;100\% \end{array}$$

### 2.8. Statistical Analysis

All experiments in this study were carried out with three parallel replicates, and all experimental data are expressed as mean ± standard deviation (mean ± SD). SPSS 22.0 software (IBM, Armonk, NY, USA) was used for statistical analysis of the data, and the significance of differences between groups was tested by one-way analysis of variance (one-way ANOVA), with the significance level set at *p* < 0.05. OriginPro 2021 software (OriginLab, Northampton, MA, USA) was adopted for data fitting (including the first-order kinetic model of TVB-N accumulation, Arrhenius equation, and linear regression of ΔE and TVB-N) and graph plotting, and the goodness of fit of the model was evaluated by the coefficient of determination (R^2^). For the validation of the shelf-life prediction model, three evaluation indicators were used: relative error (RE), mean absolute error (MAE) and root mean square error (RMSE).

## 3. Results and Discussion

### 3.1. Physicochemical Properties of Labels

A comparative analysis was conducted on the SEM, photocatalytic activity, and UV absorption characteristics of 1C-TiO_2_, 2C-TiO_2_, and 3C-TiO_2_. Only 2C-TiO_2_ exhibited a distinct particle morphology, with significantly reduced photocatalytic activity and demonstrating the strongest absorption characteristics in the UV region (Supplementary Figure S1). After comprehensive evaluation, 2C-TiO_2_ was ultimately selected as the functional component for the smart label (Supplementary Figure S2). Four types of labels—GA matrix (AG + glycerol + GG), GAB (GA + BA), GABT-0.05 (GAB + 0.05 wt% 2C-TiO_2_), and GABT-0.1 (GAB + 0.10 wt% 2C-TiO_2_)—were prepared using an agarose/gellan gum/glycerol composite matrix system with anthocyanin as the pH-responsive indicator. The effects of 2C-TiO_2_ loading on the microstructure, chemical compatibility, crystalline behavior, and thermal stability of the labels were investigated using scanning electron microscopy (SEM), Fourier transform infrared spectroscopy (FTIR), X-ray diffraction (XRD), and thermogravimetric analysis (TGA).

**Figure 1:**
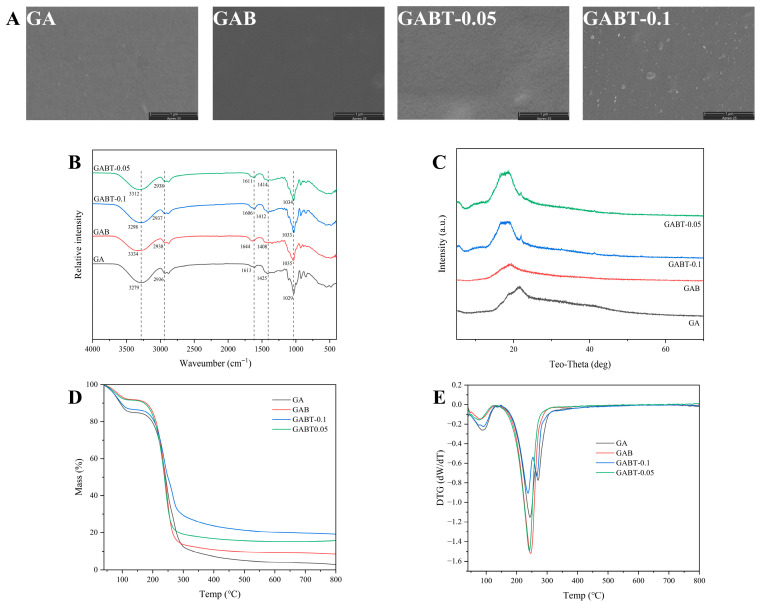
FESEM (**A**), FT-IR (**B**), XRD (**C**), TGA (**D**), and DTG (**E**) curves of GA, GAB, GABT-0.05, and GABT-0.1 labels.

SEM (Figure 1A) revealed that 2C-TiO_2_ was uniformly dispersed in GABT-0.05, while aggregation occurred in GABT-0.1. FTIR (Figure 1B) indicated that the incorporation of 2C-TiO_2_ modulated the hydrogen-bonding network (blue shift of the O-H peak), and all components exhibited good compatibility. XRD (Figure 1C) confirmed the amorphous nature of the matrix, with the intensity of the anatase TiO_2_ characteristic peak increasing with higher 2C-TiO_2_ content in the GABT series. TGA-DTG analyses (Figure 1D,E) showed that the residual mass increased from ~10% for GA to ~20% for GABT-0.1 upon addition of 2C-TiO_2_. However, the thermal decomposition kinetics exhibited a non-monotonic trend: GAB and GABT-0.05 displayed a sharp mass-loss peak at ~280 °C (~−1.5 dW/dT), whereas GA and GABT-0.1 showed broader peaks with reduced intensity. These results indicate that an appropriate amount of 2C-TiO_2_ (0.05 g) maintained thermal stability without significantly altering the decomposition behavior, while excess loading delayed the degradation process. In summary, these findings clarify the role of 2C-TiO_2_ in structural regulation and thermal stabilization of the intelligent labels, providing a basis for their application in indicator labels.

### 3.2. Physical and Mechanical Properties of Labels

Physical Properties: The data presented in Table 1 indicate that the thickness of GABT-0.05 labels decreased by 0.02 mm and MC decreased by 23.57% compared to GAB. This trend is speculated to result from the introduction of 2C-TiO_2_, which may alter the hydrophilic network structure of the composite matrix and thus promote water loss. WS showed no significant differences among GA, GAB, and GABT-0.05; however, GABT-0.1 exhibited a 5.35% reduction in WS, which can be attributed to the presence of insoluble 2C-TiO_2_. WVP exhibited regular variations: GAB showed reduced WVP compared to GA due to matrix densification via hydrogen bonding between BA and the biopolymer network (consistent with SEM observations). This is consistent with the research results of Zhan et al., who reported that anthocyanin incorporation could densify the biopolymer matrix via hydrogen bonding and thus reduce water vapor permeability [28]. Conversely, the GABT-series labels exhibited slightly increased WVP, likely caused by void formation around dispersed, non-bonded 2C-TiO_2_ particles, consistent with SEM and FTIR results.

Mechanical properties: The data presented in Table 2 reveal that GA exhibited low TS (4.53 ± 0.82 MPa) and EAB (6.41 ± 1.37%). The addition of BA, forming GAB, significantly enhanced TS (174% increase to 12.42 ± 1.21 MPa) and EAB (210% increase to 19.89 ± 0.85%), indicating improved flexibility and ductility via intermolecular interactions. Incorporating 2C-TiO_2_ maintained TS (12.93–13.72 MPa) and EAB (19.09–19.99%) at levels comparable to those of GAB, confirming good dispersion without stress concentration. Notably, GABT-0.05 achieved the relatively best mechanical performance among the GABT-series labels (TS = 13.72 ± 0.56 MPa, EAB = 19.99 ± 0.79%).

### 3.3. Color Stability and pH Sensitivity of Labels

**Figure 2:**
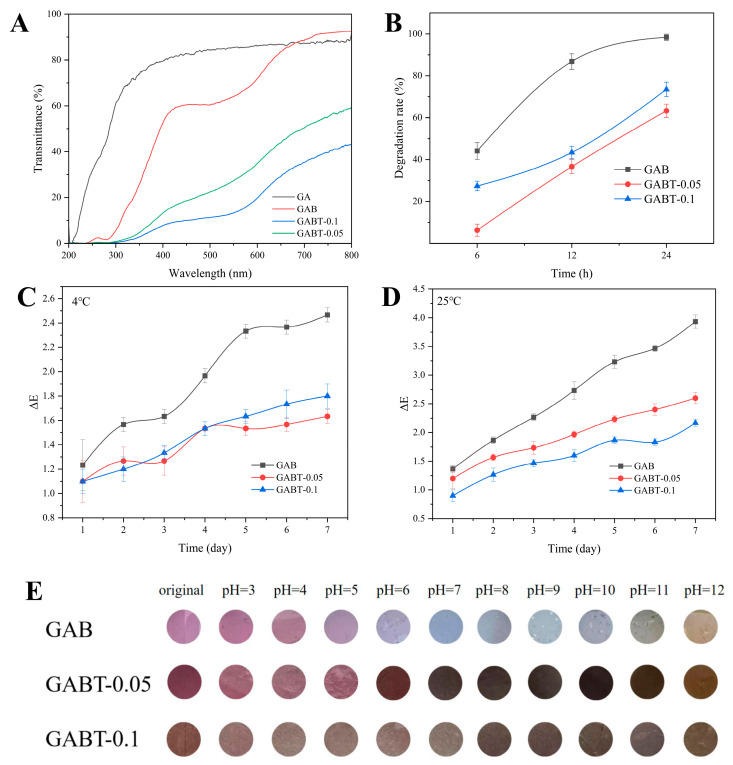
UV-Vis transmittance spectra (**A**), BA degradation rate (**B**), color stability at 4 °C (**C**) and 25 °C (**D**), and pH sensitivity (**E**) of GA, GAB, GABT-0.05, and GABT-0.1 labels.

Color stability: UV-Vis light barrier property tests revealed a transmittance gradient following GA > GAB > GABT series (Figure 2A). GAB exhibited significantly reduced transmittance due to strong UV absorption by the aromatic rings of BA [36], while the GABT series demonstrated further decreased transmittance attributed to the UV-shielding effect of 2C-TiO_2_, providing effective protection across both UV and visible light regions. The low transmittance of the label in the ultraviolet region (200–400 nm) indicates its excellent UV resistance. The photodegradation kinetics of labels (Figure 2B) indicate that the GABT-series labels exhibited markedly lower degradation rates compared to the GAB label, thus confirming the beneficial role of 2C-TiO_2_ in enhancing photostability. The GABT-0.05 label in Figure 2B exhibited the lowest BA degradation rate after ultraviolet irradiation, indicating that it retained partial color retention capability post-UV exposure, demonstrating its responsiveness to environmental changes even under UV irradiation. Storage stability experiments (Figure 2C,D) verified that the GABT labels maintained maximum ΔE values of 1.8 (4 °C) and 2.6 (25 °C) after 7 days of storage, which are significantly lower than those of GAB (2.46/3.93). An ΔE value ≤ 2 indicates “no noticeable color difference,” while 2 < ΔE ≤ 3 signifies a “mild color difference that does not affect color recognition or freshness assessment.” At 4 °C, ΔE ≤ 1.8 shows no significant color variation, whereas the maximum ΔE for GABT labels at 25 °C is 2.6, representing only a slight color difference both of these values are far below the 3.93 observed in GAB labels. These results meet the color durability requirements for freshness monitoring during shrimp storage.

pH sensitivity: The GAB label exhibits a gradient color change from light red (pH 3) to pale blue/yellow (pH 12). The GABT series also demonstrates distinct color transitions, ranging from light red (pH 3) through navy blue to pale yellow (pH 12). Notably, GABT-0.1, which contained excessive loading of 2C-TiO_2_ (0.10 wt%), showed increased color depth but slightly reduced clarity in color differences across pH values, likely due to particle aggregation induced by enhanced van der Waals forces. Ultimately, based on the chromogenic reaction and integrated with the aforementioned physicochemical performance tests, GABT-0.05 was selected as the optimal label for indicating freshness in fresh shrimp storage monitoring.

### 3.4. Shrimp Shelf-Life Prediction Model

**Figure 3:**
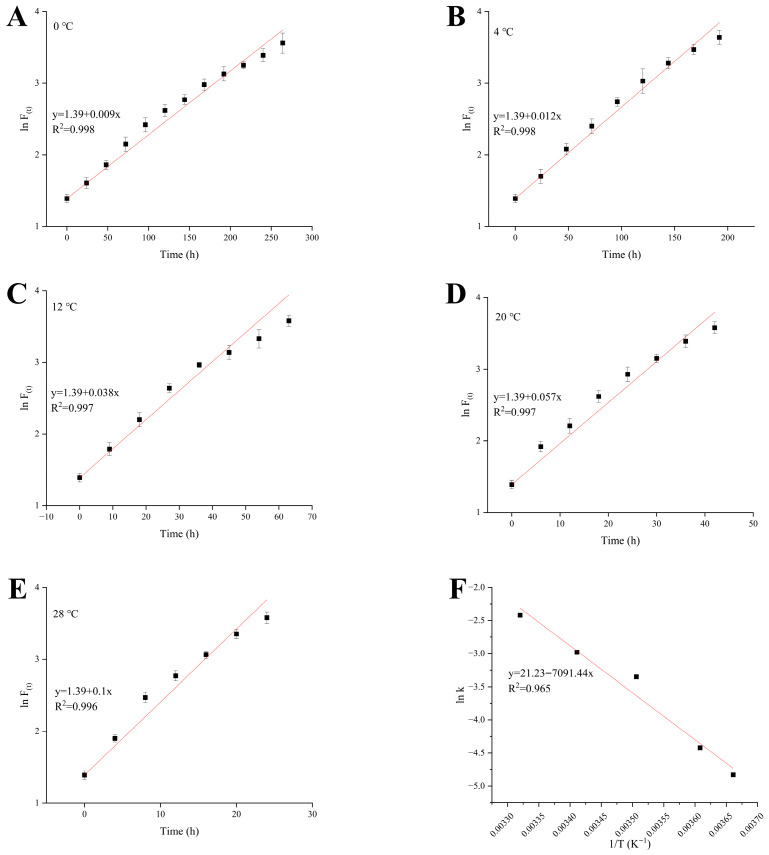
Changes in TVB-N content in shrimp during storage under different temperatures: 0 °C (**A**), 4 °C (**B**), 12 °C, (**C**) 20 °CK, (**D**), 28 °C (**E**). Linear fitting of *ln k* vs. 1/T (**F**).

The variation trend of TVB-N content in shrimp during storage is illustrated in Figure 3A–E. The determination coefficients (R^2^) of the fitting curves under all storage temperature conditions exceeded 0.96, indicating that the first-order kinetic equation effectively describes the variation of TVB-N content. The rate constants (k) for TVB-N changes were measured at 0.009, 0.012, 0.038, 0.057, and 0.089 for storage temperatures of 0 °C, 4 °C, 12 °C, 20 °C, and 28 °C, respectively. A linear regression analysis was conducted on lnk and the reciprocal of the storage thermodynamic temperature (1/T) (Figure 3F), resulting in the fitting equation y = −7091.4x + 21.23. Based on the linear characteristics of the Arrhenius logarithmic equation (lnk = (−E_a_/RT) + lnk_0_), the activation energy (Ea) for TVB-N changes was calculated to be 58,958.0 J/mol, with the pre-exponential factor (k_0_) determined to be 1.7 × 10^9^. By substituting E_a_ and k_0_ into the Arrhenius equation (k = k_0_ exp(−E_a_/RT)), the kinetic equation relating the TVB-N change rate to temperature during shrimp storage was established as k = 1.7 × 10^9^ exp(−58,958.0/RT). Furthermore, by incorporating the rate constant k and the TVB-N spoilage threshold (30 mg/100 g) into the first-order kinetic shelf-life calculation formula (t = (lnA − lnA_0_)/k), the remaining shelf-life prediction model for shrimp was derived as shown in Equation (4). This equation establishes a quantitative correlation between TVB-N content and the remaining shelf-life of shrimp under specific temperature conditions, providing a crucial theoretical basis for the development and application of intelligent labels for product shelf life based on the TVB-N index.

(4) $$\begin{array}{c} t_{RSL}=\frac{3.401-lnA_{0}}{{1.7\times 10}^{9}exp(\frac{-7091.4}{T})} \end{array}$$

where *t_RSL_* represents remaining shelf-life; *T* represents the storage temperature with the unit of absolute temperature (K); R = 8.314 J/(mol·K); ln30 = 3.401; *A*_0_ represents the current TVB-N value with the unit of mg/100 g.

### 3.5. Application of GABT-0.05 Label in Monitoring the Remaining Shelf-Life of Shrimp

**Figure 4:**
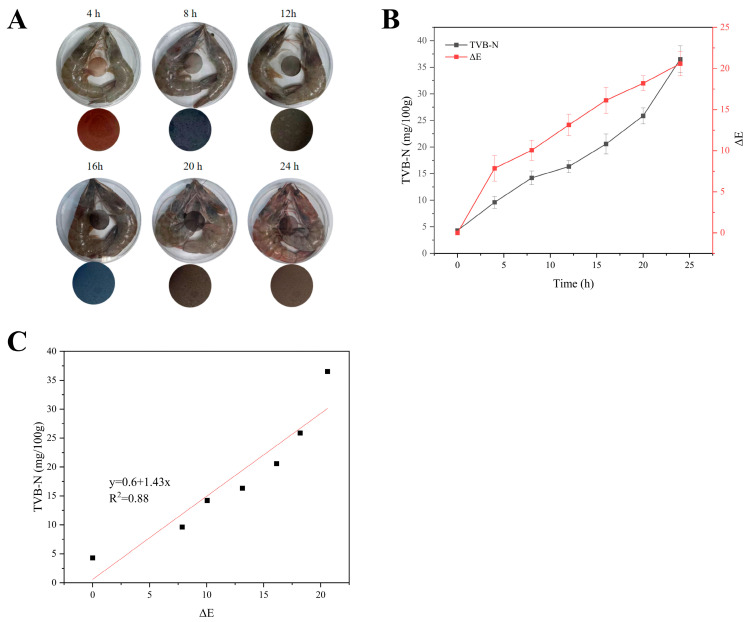
Label color change (**A**), TVB-N content (**B**), and linear fitting of TVB-N vs. Δ*E* (**C**) during shrimp storage at 25 °C.

Shelf-Life Prediction by Indicator Label: Based on the previously screened GABT-0.05 label with the optimal performance, the dynamic correlation between the label color change and TVB-N content was established by determining the color variation of the label and the corresponding TVB-N content during shrimp storage (Figure 4). During storage, the TVB-N content increased continuously, and the color of the GABT-0.05 label exhibited a gradual evolution from initially light red to blue and then to dark yellow (Figure 4A). In contrast, shrimp meat exhibited only mild reddening at 24 h, with a TVB-N value of 32 mg/100 g, which exceeded the spoilage threshold of 30 mg/100 g. At 8 h, the TVB-N value of shrimp meat reached 14.2 mg/100 g, with no significant visual changes observed, but the label’s ΔE value exceeded 10 (Figure 4B), confirming that the colorimetric indicator label outperformed visual inspection of shrimp meat appearance in terms of freshness detection sensitivity. Furthermore, ΔE of the label was correlated with the TVB-N content, and a linear regression equation y = 1.43x + 0.6 (Equation (5), where y represents TVB-N content and x represents ΔE, R^2^ = 0.88) was established (Figure 4C). This equation was substituted into the shelf-life prediction model derived in Section 3.4 (Equation (4)), and a novel shelf-life prediction model based on ΔE was finally constructed (Equation (6)).

Validation of Prediction Accuracy: To verify the practical application performance of the GABT-0.05 label in shrimp freshness monitoring and shelf-life prediction, the remaining shelf-life of shrimp at three typical storage temperatures (4 °C, 10 °C, 20 °C) and different time points was predicted based on the established ΔE-TVB-N correlation model and kinetic model, with prediction accuracy evaluated via relative error (RE), mean absolute error (MAE) and root mean square error (RMSE), as shown in Table 3. The results showed that RE between the predicted and actual measured values of the GABT-0.05 label was controlled within 10% at all time points under the three temperature conditions, falling within the acceptable error range for food shelf-life prediction; meanwhile, the label presented low MAE and RMSE values at each temperature, with the lowest values at 20 °C, indicating high prediction accuracy at room temperature and acceptable prediction error at low temperature (4 °C). These results fully confirmed the reliable predictive performance of the GABT-0.05 label’s color change for shrimp remaining shelf-life, and the combined use of RE, MAE and RMSE effectively supplemented the limitation of single indicator evaluation, comprehensively characterized the model’s prediction accuracy and uncertainty, and made the evaluation of model performance more scientific and rigorous. The practicality of the GABT-0.05 label for shrimp shelf-life prediction under different temperature conditions is key to its industrial application: its prediction error is low under the typical storage temperatures of shrimp, and its color change is moderately synchronized with the shrimp spoilage process in actual storage environments, showing obvious color changes 1–7 h earlier than the apparent spoilage of shrimp, which can provide an operable freshness judgment basis for retailers and consumers in the aquatic product supply chain. In addition, the label features simple operation, no need for professional instruments and real-time in situ monitoring, making it suitable for shrimp freshness detection in various links such as storage, transportation and sales, and endowing it with good practical application potential.

(5) $$\begin{array}{c} F_{\left(t\right)}=1.43∆E+0.6 \end{array}$$

(6) $$\begin{array}{c} t_{RSL}=\frac{3.401-\ln(1.43∆E+0.6)}{{1.7\times 10}^{9}exp(\frac{-7091.4}{T})} \end{array}$$

## 4. Conclusions

This study co-embedded 2C-TiO_2_ and BA into GA composite matrix, establishing a shrimp shelf-life prediction model using label ΔE as a key parameter. The integration of UV shielding functionality and quantitative freshness monitoring was successfully achieved. Results demonstrated that 2C-TiO_2_ significantly enhanced BA photostability: after 24 h of UV exposure, BA degradation rates decreased by 35.6% compared to control groups, effectively addressing the critical challenge of natural pigment inactivation under light conditions. Based on the correlation between ΔE and TVB-N, label color changes enabled real-time visual monitoring of shrimp freshness. Furthermore, the shelf-life prediction model developed using this correlation and kinetic response showed validation errors below 10% when applied under constant temperature conditions (4 °C, 10 °C, and 20 °C), demonstrating practical proof-of-concept value. In summary, the dual-functional colorimetric intelligent label developed in this study provides a viable solution for non-destructive real-time shrimp freshness monitoring, effectively overcoming limitations of traditional intelligent labels such as natural pigment degradation and challenges in quantitative colorimetric analysis. Future applications may involve developing compatible intelligent color acquisition and analysis systems, along with establishing temperature-calibrated quantitative models for ΔE-TVBN relationships, to unlock the label’s potential in food packaging applications.

## Figures and Tables

**Table 1:** Thickness, MC, WS, and WVP of composite labels.

Label Type	Thickness (mm)	MC (%)	WS (%)	WVP (×10^−9^ g m^−1^ s^−1^ Pa^−1^)
GA	0.25 ± 0.02 ^a^	47.75 ± 0.15 ^a^	46.14 ± 0.08 ^a^	3.12 ± 0.42 ^b^
GAB	0.23 ± 0.02 ^a^	52.61 ± 0.14 ^a^	48.2 ± 0.29 ^a^	2.50 ± 0.31 ^a^
GABT-0.05	0.21 ± 0.01 ^a^	29.04 ± 0.17 ^b^	42.85 ± 0.26 ^a^	2.76 ± 0.25 ^ab^
GABT-0.1	0.21 ± 0.02 ^a^	25.52 ± 0.24 ^b^	47.63 ± 0.06 ^a^	2.83 ± 0.44 ^ab^

Note: Values are mean ± standard deviation. Different superscript letters (a, b) within a column indicate significant differences (*p* < 0.05).

**Table 2:** Mechanical properties of composite labels.

Label Type	TS (MPa)	EAB (%)
GA	4.53 ± 0.82 ^a^	6.41 ± 1.37 ^a^
GAB	12.42 ± 1.21 ^b^	19.89 ± 0.85 ^b^
GABT-0.05	13.72 ± 0.56 ^b^	19.99 ± 0.79 ^b^
GABT-0.1	12.93 ± 0.76 ^b^	19.09 ± 0.66 ^b^

Note: Values are mean ± standard deviation. Different superscript letters (a, b) within a column indicate significant differences (*p* < 0.05).

**Table 3:** RE, MAE, and RMSE between predicted value and measured value.

Temp/°C	Time (h)	ΔE	Predicted (h)	Observed (h)	RE	MAE	RMSE
4	24	6.8	81.2	75.0	8.3%		
4	48	9.9	54.0	50.0	8.0%	4.03	4.40
4	72	14.5	25.9	24.0	7.9%		
10	10	5.3	57.6	53.0	8.7%		
10	26	8.5	37.9	36.0	5.3%	2.93	3.17
10	36	11.3	26.3	24.0	9.6%		
20	10	9.4	14.7	14.0	5.0%		
20	15	11.4	10.8	10.0	8.0%	0.63	0.66
20	20	15.3	5.4	5.0	8.0%		

Note: MAE/RMSE is the average value at the same temperature.

## Data Availability

The original contributions presented in this study are included in the article/Supplementary Material. Further inquiries can be directed to the corresponding author.

## Supplementary Materials

The following supporting information can be downloaded at: https://www.mdpi.com/article/10.3390/foods15081388/s1, Figure S1. SEM (A), FT-IR (B), XRD (C), and UV absorption (D) spectra of nano-TiO_2_ and carbon-coated nano-TiO_2_. Photocatalytic activity of different samples (E), XPS spectra of nano-TiO_2_ and 2C-TiO_2_: Survey (F), C 1s (G), O 1s (H), Ti 2p (I); Figure S2. Create infographic with labels.

## Abbreviations

AG	Agarose
BA	Blueberry anthocyanins
C-TiO_2_	Carbon-coated nano-TiO_2_
ΔE	Color difference
EAB	Elongation at break
FTIR	Fourier transform infrared spectroscopy
GA	Agarose/gellan gum/glycerol
GG	Gellan gum
MAE	Mean absolute error
MC	Moisture content
RE	Relative error
RMSE	Root mean square error
SEM	Scanning electron microscope
TGA	Thermogravimetric analysis
TS	Tensile strength
TVB-N	Total volatile basic nitrogen
UV	Ultraviolet
WVP	Water vapor permeability
WS	Water solubility
XRD	X-ray diffraction
