# Supplementary material for: Integration of UV Stability and Shelf-Life Prediction in a Colorimetric Intelligent Label for Real-Time Monitoring of Shrimp Freshness
Source: Foods. 2026 Apr 16;15(8):1388. doi: 10.3390/foods15081388 (PMC13115143; doi:10.3390/foods15081388)
Supplement: Supplementary file 1 [file foods-15-01388-s001.zip › foods-4234290-supplementary.pdf]

Supplementary Materials

Integration of UV Stability and Shelf-Life Prediction in a Colorimetric Intelligent Label for Real-Time Monitoring of Shrimp Freshness

Xiujin Chen [1,†], Shiqiang Yu [1,2,†], Yang Qu [2], Jing Wang [2], Minghui Dai [2], Weiguo Song [2], Peihong Liu [2] and Yujuan Suo [2,*]

[1] Henan International Joint Laboratory of Green Food Processing and Quality and Safety Control, College of Food and Bioengineering, Henan University of Science and Technology, Luoyang 471000, China; chenxiujin9610@126.com (X.C.); y17630964865@163.com (S.Y.)

[2] Institute for Agro-Food Standards and Testing Technology, Laboratory of Quality & Safety Risk Assessment for Agro-Products (Shanghai), Ministry of Agriculture and Rural Affairs, Shanghai Academy of Agricultural Sciences, Shanghai 201403, China; niyinluo@163.com (Y.Q.); wangjing970222@163.com (J.W.); daiminghui183@163.com (M.D.); songweiguo@saas.sh.cn (W.S.); liupeihong@saas.sh.cn (P.L.)

* Correspondence: suoyujuan@saas.sh.cn; Tel.: +86-21-6220-6056

† These authors contributed equally to this work.

1Materials characterization

1.1. Field Emission Scanning Electron Microscopy (FESEM) of C-TiO_2

In the carbon-coated nano-TiO_2 prepared with glucose, the carbon layer is thinnest when the glucose dosage is 1 g. When the glucose dosage is 2 g, the carbon layer is thicker and more uniform. However, when the glucose dosage is 3 g, the carbon layer becomes excessively thick, leading to agglomeration. Fig. S1A showed pristine nano-TiO_2 particles were smooth, clean, spherical/near-spherical, well-dispersed with clear boundaries. 1C-TiO_2 indicated thin carbon coating formed with 1g glucose under hydrothermal conditions. 2C-TiO_2 particles displayed a rough, fluffy amorphous carbon layer, obscuring edges and slightly increasing particle size (carbon layer 10-20 nm thick), improving dispersion due to steric hindrance. 3C-TiO_2 showed severe agglomeration due to excess carbon[1].

1.2. FT-IR Spectroscopy of C-TiO_2

FT-IR spectra (Fig. S1B) showed a broad peak at ~3437 cm^{-1} (O-H stretching, adsorbed H_2O/Ti-OH), slightly increasing intensity with carbon content. The peak at ~1630 cm^{-1} (H-O-H bending) intensity increased significantly with carbon content, indicating enhanced water adsorption[2] A weak peak at ~1400 cm^{-1} (C-O stretching) suggested residual oxygen-containing groups, confirming carbon coating. The strong peak below 450 cm^{-1} (Ti-O stretching) remained unchanged, confirming the TiO_2 core structure. Spectra similarity indicated carbonization occurred on the TiO_2 surface, forming a core-shell structure[3].

1.3. XRD Analysis of C-TiO_2

XRD patterns (Fig. S1C) for all samples matched anatase TiO_2 (JCPDS No. 21-1272), with main peaks at 25.3° (101), 37.8°, 48.0°, 53.9°, 55.1°, and 62.7°. A faint shoulder at 27.5° indicated trace rutile phase (JCPDS No. 21-1276). Identical patterns confirmed carbon coating did not alter the TiO_2 crystal structure[4].

1.4. UV Absorption of C-TiO_2

Nano-TiO_2 showed strong UV absorption (peak ~1.6 at ~250 nm), dropping sharply above 300 nm (Fig. S1D). Carbon coating significantly enhanced absorption across 300-800 nm compared to nano-TiO_2, attributed to sp^2 carbon structure and interfacial effects. 1C-TiO_2 showed the highest long-wavelength absorption. 3C-TiO_2 performance decreased due to excess carbon agglomeration. 2C-TiO_2 absorption was intermediate, suggesting optimal carbon loading is critical.

1.5. Photocatalytic Activity of C-TiO_2

MB degradation curves (Fig. S1E) showed minimal self-degradation. All catalysts initiated degradation under visible light. The degradation efficiency order was: 3C-TiO_2 < 2C-TiO_2 < 1C-TiO_2 < nano-TiO_2. While carbon-coated samples retained photocatalytic ability, it was suppressed compared to nano-TiO_2, with 2C-TiO_2 and 3C-TiO_2 showing the most significant inhibition and similar activity[5].

1.6. XPS Analysis of C-TiO_2

XPS survey spectra (Fig. S1F) confirmed the presence of C, O, Ti in 2C-TiO_2. High-resolution C 1s spectra (Fig. 1G) showed peaks for C-C (283.8 eV) and C-O (285.8 eV) in 2C-TiO_2, while only C-C was present in nano-TiO_2 (adsorbed organics)[6]. O 1s spectra (Fig. 1H) revealed Ti-O-Ti (529.1 eV) and Ti-OH (531.6 eV) in nano-TiO_2, while 2C-TiO_2 showed Ti-O-Ti (529.5 eV), Ti-O-C (531.2 eV), and C=O (532.6 eV)[7]. Ti 2p spectra (Fig. 1I) showed peaks for Ti $2p_3/_2$ (458.2 eV) and Ti $2p_1/_2$ (463.9 eV) in both. Small binding energy shifts in 2C-TiO_2 (<0.5 eV) indicated altered surface chemical states due to interfacial interactions (electron effects, functional group coordination, lattice stress), confirming uniform carbon coating[8].

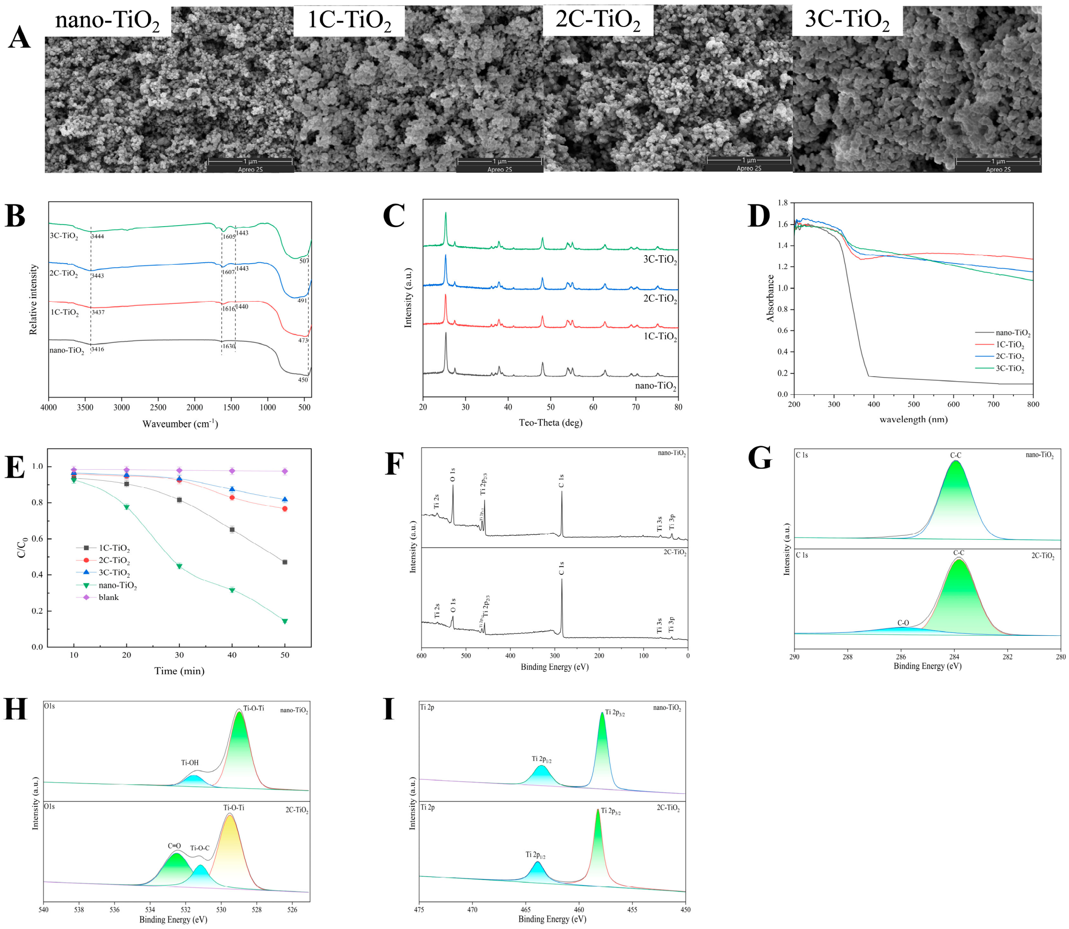

Figure S1. SEM (A), FT-IR (B), XRD (C), and UV absorption (D) spectra of nano-TiO_2 and carbon-coated nano-TiO_2. Photocatalytic activity of different samples (E), XPS spectra of nano-TiO_2 and 2C-TiO_2: Survey (F), C 1s (G), O 1s (H), Ti 2p (I).

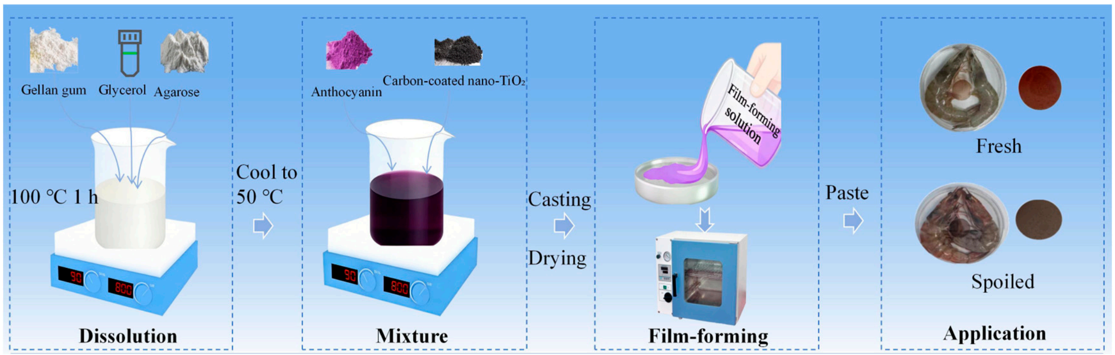

Figure S2. Create infographic with labels

Reference

1. Kumar, R., Choudhary, R., Kolay, S., Pandey, O. P., Singh, K., & Bhargava, P. (2022). Carbon coated titanium dioxide (CC-TiO_2) as an efficient material for photocatalytic degradation. *Energy Advances*, *1*(11), 926–934.
2. Li, M., Zhou, S., Zhang, Y., Chen, G., & Hong, Z. (2008). One-step solvothermal preparation of TiO_2/C composites and their visible-light photocatalytic activities. *Applied Surface Science*, *254*(13), 3762–3766.
3. Qamar, M., Yoon, C. R., Oh, H. J., Lee, N. H., Park, K., Kim, D. H., Lee, K. S., Lee, W. J., & Kim, S. J. (2008). Preparation and photocatalytic activity of nanotubes obtained from titanium dioxide. *Catalysis Today*, *131*(1), 3–14.
4. Fan, R., Zhang, F., Wang, L., Liu, Y., & Liu, J. (2026). One-step fabrication of C-modified TiO_2 composite photocatalysts for enhanced visible light photocatalytic performance. *Results in Engineering*, *29*, 108566.
5. Ali, Z., Ismail, J., Hussain, R., Shah, A., Mahmood, A., Mohammad Toufiq, A., & Rahman, S. ur. (2020). Hydrothermal synthesis and characterization of carbon-doped TiO_2 nanoparticles*. *Chinese Physics B*, *29*(11), 118102.
6. Ivanov, S., Barylyak, A., Besaha, K., Dimitrova, A., Krischok, S., Bund, A., & Bobitski, J. (2016). Enhanced lithium ion storage in TiO_2 nanoparticles, induced by sulphur and carbon co-doping. *Journal of Power Sources*, *326*, 270–278.
7. Liang, Y., Li, N., Li, F., Xu, Z., Hu, Y., Jing, M., Teng, K., Yan, X., & Shi, J. (2019). Controllable nitrogen doping and specific surface from freestanding TiO_2@carbon nanofibers as anodes for lithium ion battery. *Electrochimica Acta*, *297*, 1063–1070.
8. Kumar, R. (2025). Glucose derived carbon-coated titanium dioxide (GDCC-TiO_2) as an advanced electrode material for supercapacitor applications. *Diamond and Related Materials*, *156*, 112389.
